# Detection of Isopeptide Bonds in Monoclonal Antibody Aggregates

**DOI:** 10.1007/s11095-021-03103-y

**Published:** 2021-09-15

**Authors:** Thomas Powell, Michael J. Knight, Amanda Wood, John O’Hara, William Burkitt

**Affiliations:** Biomolecular Formulation and Characterization Sciences, UCB, Slough, SL3WE UK

**Keywords:** cross-linking, isopeptide bond, mass spectrometry, monoclonal antibody

## Abstract

**Purpose:**

A major difficulty in monoclonal antibody (mAb) therapeutic development is product aggregation. In this study, intermolecular isopeptide bonds in mAb aggregates were characterized for the first time. We aim to propose a mechanism of covalent aggregation in a model antibody using stressed studies at raised temperatures to aid in the understanding of mAb aggregation pathways.

**Methods:**

Aggregate fractions were generated using raised temperature and were purified using size-exclusion chromatography (SEC). The fractions were tryptically digested and characterized using liquid chromatography hyphenated to tandem mass-spectrometry (LC–MS/MS).

**Results:**

An increased amount of clipping between aspartic acid and proline in a solvent accessible loop in the constant heavy 2 (CH2) domain of the mAb was observed under these conditions. Detailed peptide mapping revealed 14 isopeptide bonds between aspartic acid at that cleavage site and lysine residues on adjacent antibodies. Two additional isopeptide bonds were identified between the mAb HC N-terminal glutamic acid or a separate aspartic acid to lysine residues on adjacent antibodies.

**Conclusions:**

Inter-protein isopeptide bonds between the side chains of acidic amino acids (aspartate and glutamate) and lysine were characterized for the first time in mAb aggregates. A chemical mechanism was presented whereby spontaneous isopeptide bond formation could be facilitated via either the aspartic acid side chain or C-terminus.

**Supplementary Information:**

The online version contains supplementary material available at 10.1007/s11095-021-03103-y.

## Introduction

Monoclonal antibodies (mAbs) have become the most important form of protein therapeutic and represent five of the top ten selling drugs by revenue worldwide (1). Despite the many advantages of antibodies as therapeutics, such as their high target specificity and being generally well tolerated (2, 3); a difficulty in the development of mAbs is aggregation (4, 5). Aggregates can negatively impact the activity of the drug, as well as potentially induce immunogenic responses. The difficulty in avoiding these conditions is that each mAb has unique and different mechanisms of degradation.

Aggregation can be affected through a variety of mechanisms, involving both covalent and non-covalent bonds (6–8). Aggregates can be broadly categorized into different classes (9, 10) including reversible and non-reversible non-covalent small oligomers (e.g. dimers, trimers etc.); covalent small oligomers as well as submicron; subvisible (50-3000 nm) and visible aggregates.

Aggregates held together by covalent bonds, or cross-links, can be further subdivided into non-reducible and reducible aggregates, i.e. those formed through the formation of disulfide bonds. Although non-reducible cross-linked structures have previously been reported in mAbs (11, 12), the identification of novel covalent aggregation pathways is challenging due to a lack of efficient tools in identifying cross-links with an unknown structure and therefore unknown masses. Consequently, undefined species identified via techniques such as mass spectrometry are difficult to identify via automated search engines. Indeed, many mechanisms of covalent aggregation remain elusive.

Antibody aggregation is understood to progress via several parallel and competing pathways (13, 14). Whilst each pathway progresses differently, the formation of dimers is understood as one of the early steps of aggregation. This can progress through several mechanisms. For example, via the partial unfolding of a monomer leading to a more aggregation prone structure. Or alternatively the association of two natively folded monomers.

Furthermore, since dimers are typically the major aggregate present in mAbs, their characterization is of particular interest. The localization of the interface sites can be driven by both amino acid sequence and higher order structure of the therapeutic. There are examples of dimer formation being driven by Fab-Fab, Fc-Fc and Fab-Fc interactions (15–19). However, the behavior of dimers with regards to driving aggregation of mAbs is poorly understood. It is therefore of high importance to understand the structure and mechanism of the different dimer species present.

Liquid chromatography tandem mass-spectrometry (LC–MS/MS) has become the method of choice for the characterization of mAb degradants over the past decades (20, 21). Peptide mapping is a proven technique for determining primary sequence information as well as the identification and quantification of post-translational modifications (PTMs) (22), particularly with the advent of high-resolution accurate-mass (HRAM) MS (23).

LC–MS/MS has previously been used in the characterization of novel cross-links in mAb aggregates. Liu *et al*. used this technique to characterize intra-molecular cross-links between conserved histidine residues within an IgG1 hinge region (24). Furthermore, Xu* et al*. identified inter-molecular cross-links between histidine, lysine and cysteine residues (25).

Forced degradation studies have become an integral part of mAb development. These provide an opportunity to understand major degradation pathways not observed during stability studies (26). Forced degradation may be performed at elevated temperatures exceeding the nominal storage conditions, with the aim of generating substantial levels of degradation within a short time period. High temperature stress is proven to be efficient at causing both covalent and non-covalent aggregate formation (27, 28). Incubation at increased temperatures can also accelerate degradation through peptide bond cleavage or clipping (29, 30).

In this study mAb aggregates were generated using elevated temperatures and isolated via size-exclusion chromatography. The enriched HMW fractions of the IgG4 mAb were tryptically digested and analyzed via LC MS/MS. Detailed peptide mapping revealed a hypothesized inter-molecular isopeptide bond was formed between aspartic acid and lysine residues of two distinct antibodies. Furthermore, we present a mechanism of isopeptide bond formation which is accelerated through increased clipping in the CH2 domain of the mAb. To the best of our knowledge, our work reported herein demonstrates a novel mechanism of mAb aggregation. Additionally, we are able to characterize isopeptide bonds in IgG1 formats, which together with IgG4 account for the majority of mAb based therapeutics. We expect isopeptide bond formation to be probable in other IgG formats where the Asp-Pro bond in the CH2 domain can be easily cleaved, such as IgG2.

## Materials and Methods

### mAb Formulation

mAbA (IgG4) and mAbB (IgG1) were produced in Chinese hamster ovary (CHO) cells, formulated at concentrations of 140 and 160 mg mL^−1^ in pharmaceutically relevant formulation buffers (specific components are proprietary). Protein concentration was determined using a nanodrop spectrophotometer. The absorbance at 280 nm was converted to concentration in mg/ml using theoretically pre-determined extinction coefficients using amino acid sequence.

## Generation of Stressed Sample

mAbA was incubated at a temperature of 37°C or 50°C for 3, 7, 14 or 28 days and subsequently stored at -70°C prior to analysis.

### Aggregates by Size Exclusion Chromatography

Preparative size exclusion chromatography was performed on an Akta system using a HiLoad 26/600 prep grade column (mobile phase 0.1 M Sodium Phosphate (NaP), 0.1 M Sodium Chloride (NaCl), pH 7.0). Fractions containing monomer and dimer were pooled and concentrated using centrifugal concentrators with a MWCO of 10 kDa. Samples were stored at -70°C before analysis.

Analytical size exclusion chromatography was performed using an Agilent HPLC and an Xbridge Protein BEH SEC 200 Å (7.8 × 300 mm, 3.5 µm) column (mobile phase 0.1 M NaP, 0.1 M NaCl, pH 7.0). Separation was performed at a flow rate of 0.5 ml min^−1^ and a load mass of 100 µg.

### SDS-PAGE

SDS-PAGE analysis was performed using 4–12% Bis–Tris gels and the SeeBlue™ Plus2 Pre-stained Protein Standard (*Invitrogen*). For reduced analysis, NuPAGE™ Sample Reducing Agent was added to the sample. All samples were incubated at 80°C for 5 min prior to analysis. Gels were stained using SimplyBlue™ SafeStain. Densitrometry was performed using ImageLab software using manual band detection.

## Peptide Mapping

### Liquid Chromatography

80 μg of mAbA was reduced using dithiothreitol, alkylated with iodoacetamide and tryptically digested. Peptides were separated using online reversed phase LC (Dionex UltiMate 3000), using a binary solvent system consisting of mobile phase A (water ((PURELAB Ultra, ELGA, High Wycombe, UK)) with 0.1% formic acid (Thermo Fisher Scientific,)) and mobile phase B (acetonitrile (ROMIL, Cambridge, UK) with 0.1% formic acid (Thermo Fisher Scientific)). Peptides were loaded onto an ACQUITY UPLC BEH C_18_ Column (130 Å, 1.7 µm, 2.1 mm × 150 mm) (Waters, Milford, MA, USA). Peptides were injected in mobile phase A and separated over a linear gradient from 1 to 36% mobile phase B with a flow rate of 200 µL min^−1^. Peptide separation was performed over a period of 35 min. Samples eluted directly via a Heated Electrospray Source into the mass spectrometer.

### Mass Spectrometry

All mass spectrometry experiments were performed on a Q Exactive Plus (Thermo Fisher Scientific). Data acquisition was controlled by Xcalibur 2.1 (Thermo Fisher Scientific).

The mass spectrometer performed full MS scans (*m/z* 200—2000). Survey scans were acquired in the orbitrap with a resolution of 17,500 at *m/z* 200. Automatic gain control (AGC) target for the survey scans was 3 × 10^6^ charges with maximum injection time of 100 ms. Higher energy collisional dissociation (HCD) was performed in the HCD cell at normalized collision energy of 30%. Fragments were detected in the orbitrap at a resolution of 15,000 at *m/z* 200. Width of the precursor isolation window was 2 Th. AGC target was 1 × 10^5^ charges with a maximum injection time of 200 ms.

### Intact Mass Analysis

Samples were deglycosylated using N-glycanase and reduced using DTT. Samples were analyzed on a Synapt G2-S mass spectrometer via LC MS using a binary solvent system consisting of mobile phase A (water with 0.1% formic acid and mobile phase B (acetonitrile with 0.1% formic acid).

## Results and Discussion

**Fig. 1:**
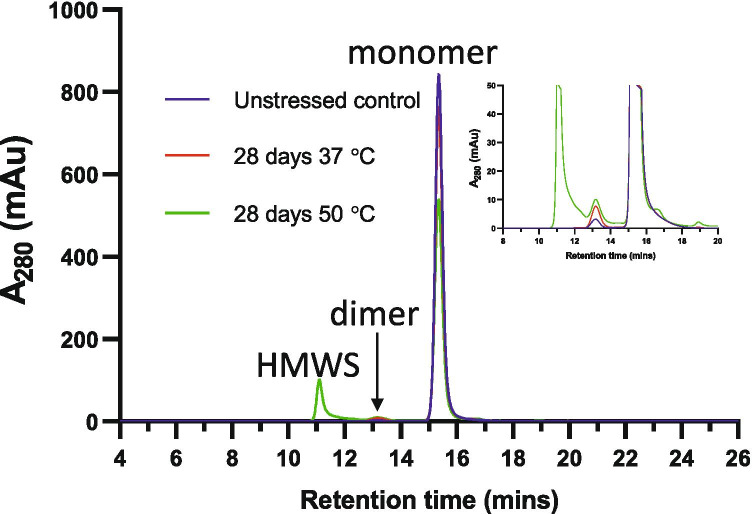
Analytical SEC of mAbA after exposure to temperature stress. Zoomed view in inset.

Figure 1 shows the analytical size exclusion chromatograms for mAbA after incubation for 28 days at 37 and 50°C. As expected, incubation of mAbA at elevated temperature led to significant aggregation, where both dimer and higher molecular weight species (HMWS) peaks were observed. Aggregate formation was more significant at 50°C than at 37°C.

### Identification of Isopeptide Bond Linked Peptides

To elucidate novel covalent mechanisms of aggregation the SEC fractions were tryptically digested. The resulting peptides were separated by liquid chromatography and analyzed using tandem mass spectrometry. Tryptic digestion of mAbA will theoretically result in 38 heavy chain peptides and 21 light chain peptides. Sequences in variable regions have remained disclosed. Tryptic heavy chain peptide 1 will be referred to as HC T01. This information is summarized in Table S1 along with the domain of each residue. Not all peptides will be detected as fully cleaved. In the case of a missed cleavage for example, heavy chain peptides 15 to 16 may also be referred to as HC T15-16.

First, a list of all fragmentation events from the LC–MS/MS analysis of the dimer fraction was interrogated and compared against the same analysis of the monomer fraction. The most abundant multiply charged species were manually verified using b and y ion assignment from HCD MS/MS data as well as each ions monoisotopic mass.

One of the most abundant ions that did not correspond to a predicted tryptic peptide was observed at *m/z* 838.8880. This ion was observed in both the aggregate and monomer fractions. Annotation of the HCD MS/MS spectra at this *m/z* showed this ion to be TPEVTCVVVDVSQED (HC T20 with a non-specific cleavage site). This peptide will be termed HC T20* for the remainder of this paper.

Strikingly, during the analysis of the HCD MS/MS data from many of the multiply charged ions in the isolated aggregates, b and y ions that were present in the fragmentation of HC20* were also observed, despite the differing *m/z* of the precursor ions.

**Fig. 2:**
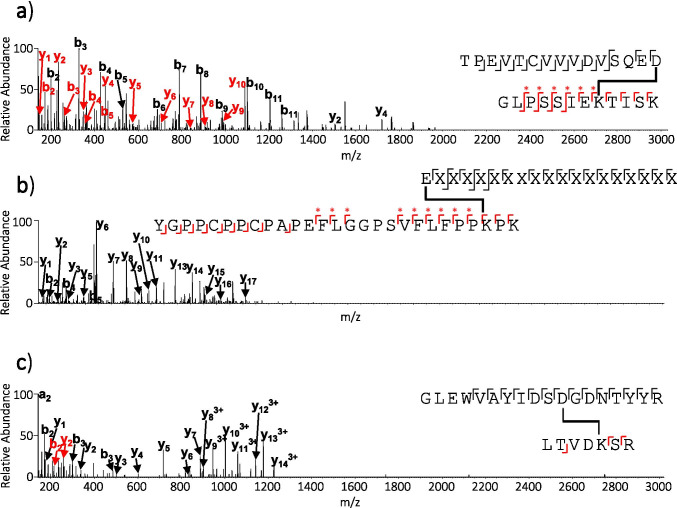
HCD MS/MS spectra for (**a**) m/z 973.1362, (**b**) HC T01 cross-linking to HC T18 and (**c**) m/z 734.8573. b and y ions are shown in black or red dependent on their corresponding peptide. * indicates cleavage of the isopeptide bond during fragmentation. Lack of label on the x-axis of Figure in 1b is due to the ion containing a proprietary amino acid sequence.

For example, an ion was observed in the aggregate fraction at *m/z* 973.1362. This ion was not present in the monomer fraction. Figure 2a shows the annotated HCD MS/MS spectrum for this ion. Interestingly, fragment ions corresponding to both HC T20* as well as peptide HC T27-28 were noted. The monoisotopic mass of the ion was a -18.01 Da mass shift from the combined theoretical mass of the two peptides. These data suggest the two peptides became covalently bonded via a dehydration reaction.

Manual analysis of the MS/MS spectrum showed that the two peptides became linked via the C-terminal aspartic acid residue of HC T20* and a lysine residue (position 8 in the peptide) in HC T27-28 (peak assignments are shown in Table S2). Under these fragmentation conditions the covalent bond between the two peptides was partially maintained, in keeping with previous data collected on covalent cross-links (31, 32). These data infer this cross-link is an isopeptide bond, i.e. a bond formed through a dehydration reaction between a hydroxy group of aspartic acid and the amino group of lysine.

It is of note that due to the ion containing two covalently linked peptides some MS/MS fragmentation patterns are difficult to predict. Numerous fragment ions in the + 2 and + 3 charge states between *m/z* ~ 1300–2000 have remained unassigned.

Next, the extracted MS peak list was searched against a peptide library containing all possible combinations of mAbA peptides with a -18.01 Da mass shift. Table 1 lists the peptides identified using this method. Each assignment could be confirmed using a run of four consecutive b or y ions from either of the theorized peptides in the ion. Of the 16 peptides listed in this table, 14 were attributed to HC20* cross-linking to other tryptic peptides via lysine. None of these isopeptide-linked peptides could be identified in the corresponding monomer fraction. When interrogating the HMWS fraction, each of the isopeptide-linked peptides from the dimer could be identified, though no additional peptides were observed.

**Table 1:** Summary of Cross-Linked Peptides Obtained Following Tryptic Digestion of IgG4

Peptide 1			Peptide 2						
Sequence	Location	Peptide	Sequence	Location	Peptide	m/z	z	RT	ΔPPM
TPEVTCVVVDVSQED*	CH2	HC T20*	VDKR	CH1	HC T15-16	725.6885	3	18.2	-4.1
TPEVTCVVVDVSQED*	CH2	HC T20*	TISKAK	CH2	HC T28-29	769.0584	3	18.5	0.6
TPEVTCVVVDVSQED*	CH2	HC T20*	AKGQPR	CH2/CH3	HC T29-30	772.0498	3	18.2	0.2
TPEVTCVVVDVSQED*	CH2	HC T20*	X	VH	HC T05-06	X	X	X	1.8
TPEVTCVVVDVSQED*	CH2	HC T20*	EYKCK	CH2	HC T24-25	795.7031	3	18.3	-0.1
TPEVTCVVVDVSQED*	CH2	HC T20*	CKVSNK	CH2	HC T25-26	798.3826	3	18.3	0.4
TPEVTCVVVDVSQED*	CH2	HC T20*	LTVDKSR	CH3	HC T35-36	826.0794	3	19.5	0.0
TPEVTCVVVDVSQED*	CH2	HC T20*	VSNKGLPSSIEK	CH2	HC T26-27	972.8196	3	20.5	-2.2
TPEVTCVVVDVSQED*	CH2	HC T20*	GLPSSIEKTISK	CH2	HC T27-28	973.1632	3	21.9	1.4
LTVDKSR	CH3	HC T35-36	X	VH	HC T44	X	X	X	0.4
TPEVTCVVVDVSQED*	CH2	HC T20*	TYTCNVDHKPSNTK	CH1	HC T14	831.3844	4	17.6	-1.4
TPEVTCVVVDVSQED*	CH2	HC T20*	X	VH	HC T01	X	X	X	1.1
TPEVTCVVVDVSQED*	CH2	HC T20*	X	VL	LC T01	X	X	X	1.0
TPEVTCVVVDVSQED*	CH2	HC T20*	SSQSLVGASGKTYLYWLFQKPGK	VL	LC T3-4	1051.5306	4	26.5	1.2
TPEVTCVVVDVSQED*	CH2	HC T20*	YGPPCPPCPAPEFLGGPSVFLFPPKPK	Hinge/CH2	HC T18	1153.5614	4	27.9	-1.0
X	VH	HC T01	YGPPCPPCPAPEFLGGPSVFLFPPKPK	Hinge/CH2	HC T18	X	X	X	3.0

The first of the two reported cross-linked peptides that did not involve HC20* was predicted to correspond to HC T01 cross-linking to HC T18 via an isopeptide bond. Figure 2b shows the HCD MS/MS data for this ion. B and y ion assignment confirmed this and showed that the isopeptide bond was formed between the N-terminal glutamic acid of HC T01 and the lysine residue in position 26 of HC T18 (see Table S3 for a full list of ion assignments). Here, the glutamic acid residue is also the protein N-terminus and is in an extremely solvent accessible region of the antibody. Note, sequence information in variable parts of the mAb are proprietary and amino acids and corresponding mass values are represented as X.

The second of the reported isopeptide linked peptides not involving HC20* was reported at *m/z* 734.8573. This ion was shown to correspond to aspartic acid in HC T04 cross-linking to a lysine residue in HC T35-36 (see Fig. 2c for annotated HCD MS/MS data and Table S4 for ion assignments). Note, only the most abundant fragment ions are annotated here.

### Isopeptide Bond Dynamics

**Fig. 3:**
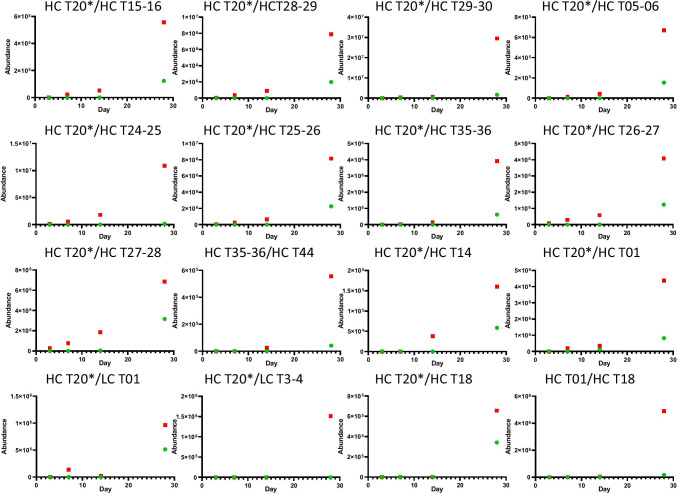
MS signal intensity of each isopeptide linked peptides in mAbA for incubations at 37°C (green) or 50°C (red) for 3, 7, 14 days and 28 days.

To monitor the formation of isopeptide bonds mAbA was incubated at 37°C or 50°C for 3, 7, 14 and 28 days. Figure 3 shows the MS signal intensity of each of the isopeptide linked peptides listed in Table 1. The MS signal has been normalized against the relative load of each LC–MS analysis. A positive correlation between the abundance of each cross-linked peptide and increased incubation time and temperature was noted, with the sharpest increase occurring between 14 and 28 days at both temperature points (Fig. 3).

Next, % HMWS as determined by SEC analysis was plotted for the different incubation temperatures (Figure S1). This mirrors the relative increases observed with isopeptide bond formation (Fig. 3). However, we expect there to be several parallel pathways of mAbA aggregation at elevated temperature; including non-covalent aggregation driven by partial unfolding and intermolecular disulphide bond formation. Indeed, our SDS-PAGE analysis confirms that a significant proportion of the aggregates are non-covalent or reducible (Figure S2). The identification of isopeptide bond formation represents the discovery of a new pathway among several competing mechanisms of aggregation. Note, the incubation temperatures of 37 and 50°C are below the Tm of mAbA (Tm1 ~ 61°C, Tm2 ~ 73°C) and so we do not expect significant unfolding to occur at these temperatures.

The dimer species from the 28 day stressed samples (as well as from an unstressed control) were isolated using preparative size exclusion chromatography and analysed by SDS-PAGE (Figure S2). The dimer samples contained a significant amount of SDS-resistant dimer (both unstressed and stressed fractions), suggesting a significant proportion of the dimer species are covalently linked. Analysis under reduced conditions showed that the majority of the covalent dimer was reducible. However, each of the dimer samples contained some non-reducible covalent HMWS. These bands were more intense in the temperature stressed dimers; densitometry analysis reveals that these non-reducible species account for approximately 10% of the total band intensity in the temperature stressed dimer samples. This suggests a reason why the higher mass species are not observed in the reduced mass analysis. Investigations into the nature of the mechanisms of reducible aggregates will be addressed in a separate study.

**Fig. 4:**
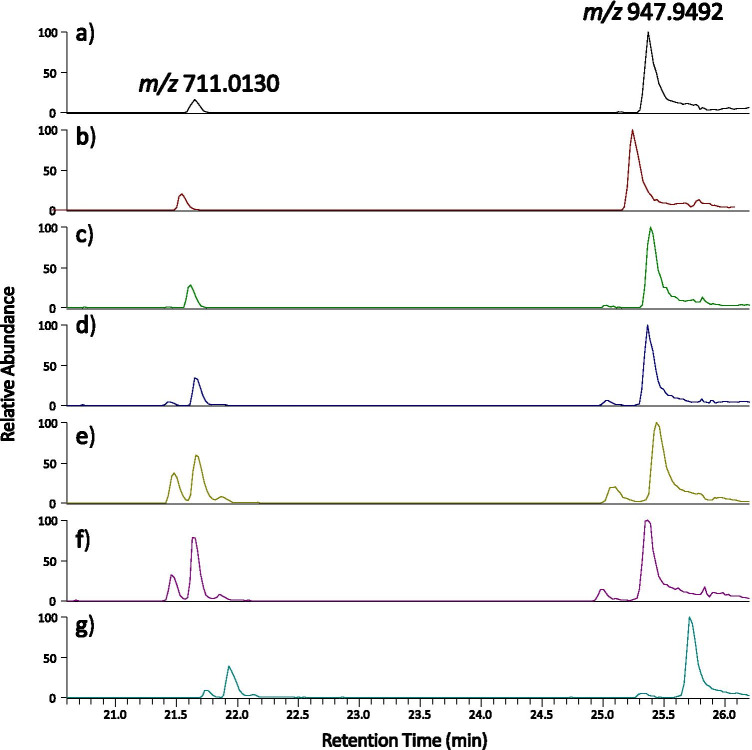
Extracted ion chromatogram at m/z 711.0130 and 947.9492 for mAbA (**a**) unstressed as well as incubated at 50°C for (**b**) 3, (**c**)7, (**d**)14, (**e**)28 days and isolated (**f**) dimer and (**g**) monomer from 28 day incubation.

Figure 4 shows the extracted ion chromatogram (EIC) for peptide HC T20 (4 + at *m/z* 947.9492) and the C-terminal fragment of HC T20 (3 + at *m/z* 711.0130) following Asp-Pro clipping (PEVQFNWYVDGVEVHNAK) at their most abundant charge states for samples incubated at 3, 7, 14 and 28 days at 50°C. There is a positive correlation between the amount of clipping at this site and incubation time and temperature.

It is noted that the introduction of a second chromatographic peak for both *m/z* values at the highest incubation times is observed. This is theorized to be due to the isomerization of aspartic acid, which is an amino acid present in both peptides. This degradation modification has previously been shown to occur in mAbs, due to the acidic nature of formulation buffers (33), and is known to be accelerated by heat (34).

The EICs of the same *m/z* values for the aggregate and monomer fractions from the SEC analysis of the samples incubated at 28 days at 50°C are shown in Fig. 4f and g. There is substantially more Asp-Pro clipping in the aggregate fraction. Clipping at this site in the heavy chain has previously been hypothesized as an important factor in antibody aggregation (6), which is supported in our results. It is however, difficult to determine whether this is the direct cause of aggregation or whether the isopeptide bonds are important in stabilizing aggregates. Further experiments are required to test this, which goes beyond the scope of this study.

Next, the aggregate fraction from the samples incubated at 50°C for 28 days was reduced, deglycosylated and analysed via LC MS with no tryptic digestion. Figure S3 shows the deconvoluted MS spectra of this sample. Here, the two most abundant ions were the mAb light and heavy chains. The next most abundant ion was the heavy chain C-terminal fragment as a result of the clipping at the C-term of the CH2 aspartic acid, confirming this as the most common cleavage site. The mass corresponding to the heavy chain C-terminal fragment at the same clipping site was not observed.

We hypothesize this is due to the formation of the isopeptide bonds that we were able to identify and characterize. However, it is of note that we are not able to observe any masses when interrogating a higher mass range corresponding to this heavy chain C-terminal fragment bound to either a light or heavy chain. This suggests additional aggregation and /or clipping reactions are occurring that are not characterized in this study.

Clipping next to the C-terminal of aspartic acid has been established as the most frequent site of intra-protein clipping in mAbs (35). The mechanism of this reaction has previously been described in detail (36). Under acidic conditions, the ionized carboxylate group of aspartic acid performs nucleophilic attack on the carbonyl carbon of the peptide bond, which is subsequently broken (37). Of particular interest is that in comparison to the other Asp-Xaa bonds the Asp-Pro bond is between 8–20 times more liable. This is theorized to be due to the additional basicity of the proline side chain, which is unique amongst the other amino acid side chains in that it is integrated into the protein backbone (38).

The specific site of clipping in the IgG CH2 domain seen in this study has previously been reported as the most labile clipping site in mAbs (6). Van Buren *et al*. reported the CH2 domain becomes increasingly unstable during acidic pH, and speculated local unfolding of this region occurs under incubation at a raised temperature. In that study, IgG aggregates generated by long-term incubation at pH3 were interrogated by LC–MS. The authors hypothesized the solvent exposure of the buried hydrophobic residues in the CH2 could make it a region that especially prone to aggregation and hypothesized clipping at the Asp-Pro site was important in the mechanism of covalent aggregation, without presenting a potential mechanism. We expect the isopeptide bond discovered in our study is responsible.

In another study Perico *et al.* investigated the influence of temperature on pH induced aggregation. The authors observed a temperature-dependence of clipping and suggested that clip-mediated aggregation was responsible for increased higher order aggregates at low pH and elevated temperatures (39). However, the sites of clipping were not characterized.

Since, the described isopeptide reactions here are facilitated by the Aps-Pro clip, we expect the weakly acidic pH of most pharmaceutical formulations to be likely conditions in which these inter-protein bonds can form.

### Mechanism of Isopeptide Bond Formation

Both non-covalent and covalent interactions have been reported between the side chains of aspartic acid and lysine (40, 41). Non-covalent interactions include salt bridges which form as a consequence of hydrogen bonds between these oppositely charged amino acids, often in solvent exposed regions of the protein (42, 43).

The opposing side chain chemistry of these residues has been exploited to artificially introduce chemical cross-links. 1-Ethyl-3-(3-Dimethylaminopropyl) Carbodiimide (EDC) is a zero-length chemical crosslinker that induces coupling of the carboxyl groups (side chains of aspartic or glutamic acid) to primary amines (side chains of lysine or arginine) (44). This approach, coupled to LC MS/MS has been used to provide a measure of the proximity of amino acid residues within a protein quaternary structure, thus offering information on folding and topology (45).

Outside of artificial chemically induced cross-links, isopeptide bonds have previously been reported in biological systems, for example in ubiquitinylation, sumoylation and transglutamination reactions (46, 47). Intra-molecular isopeptide bonds have been well studied in bacteria (48). Autocatalytic reactions between the Lys ε-amino group and the carboxamide group of an Asn side chain (49) or the carboxylate group of an Asp side chain (50) have been shown to occur.

Moreover, in a recent study Friedrich and co-authors demonstrated isopeptide bond formation between aspartate and lysine residues in model peptides, predicting this is a reaction that can spontaneously occur in aged proteins (51).

The mechanisms of intramolecular isopeptide bond formation have previously been reviewed (48). Kang *et al*. summarized that in all reported isopeptide bonds between lysine and aspartic acid residues, the reaction is catalyzed by a negatively charged amino acid side chain in close proximity to the bond. In our study, where inter-molecular isopeptide bonds were formed between the side chain of aspartic acid and the side chain of lysine, we predict that the reaction is catalyzed by the aspartic acid C-terminus. Conversely, we also predict a reaction where the cross-link is formed between lysine and the aspartic acid C-terminus and the reaction is catalyzed by the Aspartic acid side chain.

**Scheme 1:**
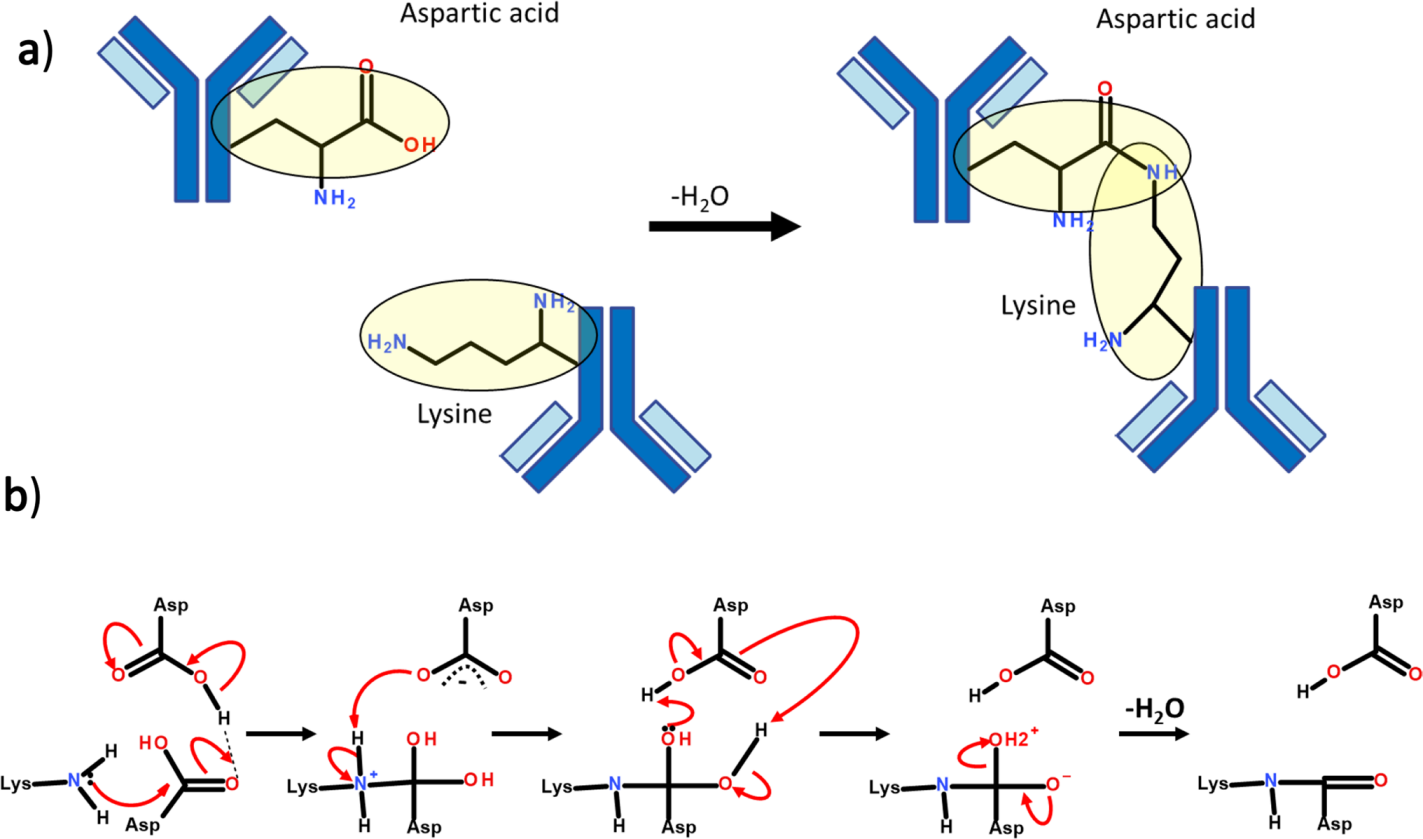
Intermolecular isopeptide bond formation (**a**) overall reaction pathway, (**b**) proposed mechanism via either side chain or C-terminal catalysis.

The reaction mechanism reviewed by Kang *et al*. is adapted in Scheme 1. Here, we predict an unprotonated amino group on the lysine residue performs nucleophilic attack on the γ-carbon of aspartic acid. A proton is then shuttled from the lysine ε-amino group to the leaving group via the nearby aspartic acid C-terminus. The protonated terminus may further accelerate catalysis by polarizing the carbonyl carbon of aspartic acid. We predict a second reaction where the isopepide bond is instead formed from the aspartic acid C-terminus.

Given their immediate proximity we hypothesise it is most likely the reactions are catalyzed by the aspartic acid side chain/terminal, although it could also be accelerated by the adjacent glutamic acid side chain.

**Fig. 5:**
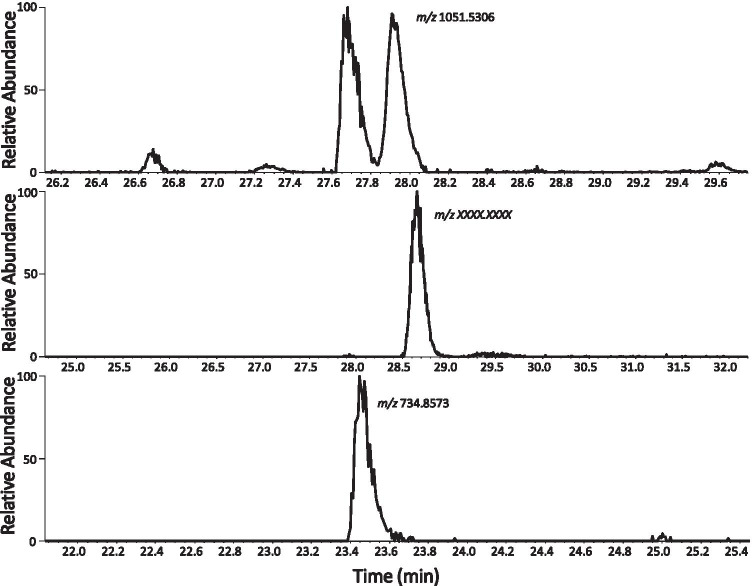
Extracted ion chromatograms for ions at m/z (**a**) 1051.5106, (**b**) (HC T01—HC T18) and (**c**) 734.8573.

Figure 5a shows the EIC for the isopeptide linked peptides HC T20* and LC T03-04 at *m/z* 1051.5106. Two distinct elution peaks were observed of comparable intensity. The MS/MS data was indistinguishable at each retention time. These data suggest two different structures for the linked peptides exist. We predict this is due a conformational change, i.e. isopeptide bond formation from the aspartic acid C-terminal or the side chain.

The EICs for the isopeptide linked peptides HC01/ HC T18 and HC T04/ HC T35-36 at *m/z* 734.8573 is shown in Fig. 5b and c. For these peptides only one chromatographic peak is observed, where bond formation between only side chains was possible. In Fig. 5b the isopeptide bond is formed between glutamic rather than aspartic acid. In Fig. 5c the aspartic acid site of bond formation is not situated on a peptide terminus.

All isopeptide linked peptides identified in Table 1 could be identified in the aggregate fractions but not in the monomer fraction. Moreover, isopeptide bonds were observed in all domains across the antibody. Given this random distribution we consider it most likely that the isopeptide bonds are inter-protein and present a mechanism of direct covalent aggregation. However, we acknowledge there is a possibility bonds may be intra-protein and indirectly favor aggregation by contributing towards conformational instability of the molecule.

We also acknowledge an alternate hypothesis for the observation of isopeptide bonds in dimer fractions. Potentially we may have identified regions of the antibody that are prone to interface during a non-covalent aggregation event. The stabilisation between other residues at these regions may facilitate isopeptide bond formation. Under this assumption, rather than initiating the beginning of an aggregation pathway, isopeptide bonds may instead be instrumental in stabilizing the aggregate form leading to HMWS.

## Conclusion

The primary goal of this work was to gain an understanding of novel covalent aggregation pathways in mAbs. The data presented here provides a potential aggregation mechanism in an IgG4. This involves clipping at the C-term of aspartic acid in the CH2 domain followed by subsequent isopeptide bond formation between a lysine residue on an adjacent antibody. This was observed under stressed studies at a high temperature.

This mechanism of action is not exclusive to IgG4. Previous work in our laboratory on other mAbs was revisited and aggregation via this pathway was observed in other IgG formats. As an example, Figure S4 shows an annotated HCD of a cross-link formed through an identical mechanism in an IgG1 (mAbB). The formulation buffers used for these two different formats were both pharmaceutically relevant, but at differing pHs with some distinctions in components. Despite this isopeptide bonds were identified in both. We expect isopeptide bond formation to be probable in other IgG formats where the Asp-Pro bond in the CH2 domain can be easily cleaved, such as IgG2.

Whilst this data presents a documentation of isopeptide bond formation in mAb aggregates, we expect other mechanisms of covalent aggregation to exist. With more sensitive tools of detection as well as the development of more sophisticated software, additional mechanisms of covalent aggregation are likely to be presented in the future. This will be explored in future work.

## Supplementary Information

Below is the link to the electronic supplementary material.

Supplementary file1 (PPTX 1007 kb)
