# Supplementary material for: Detection of Isopeptide Bonds in Monoclonal Antibody Aggregates
Source: Pharm Res. 2021 Sep 15;38(9):1519–30. doi: 10.1007/s11095-021-03103-y (PMC8497302; doi:10.1007/s11095-021-03103-y)
Supplement: Supplementary file 1 — Supplementary file1 (PPTX 1007 kb) [file 11095_2021_3103_MOESM1_ESM.pptx]

## Slide 1
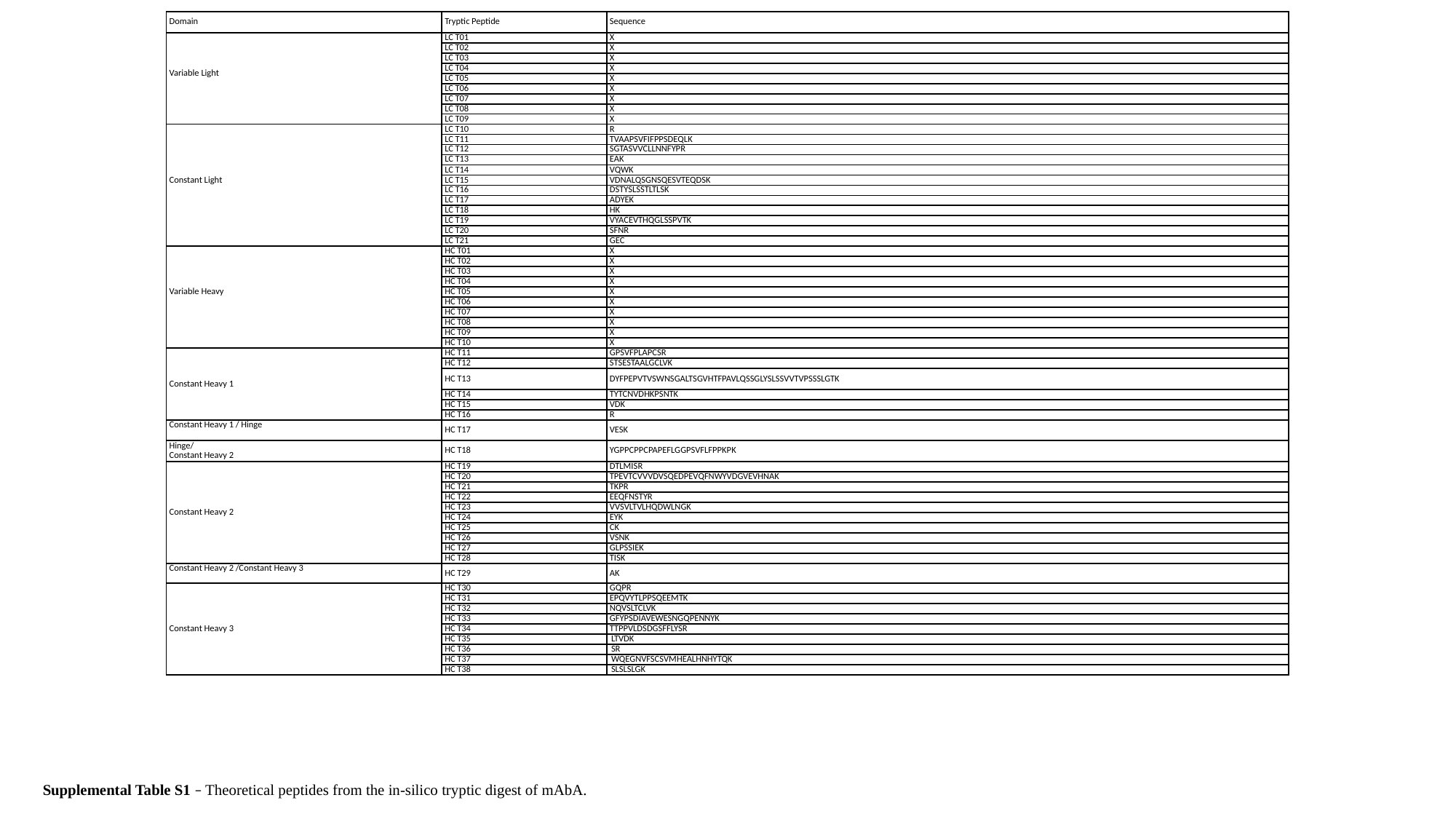

| Domain | Tryptic Peptide | Sequence |
| --- | --- | --- |
| Variable Light | LC T01 | X |
| | LC T02 | X |
| | LC T03 | X |
| | LC T04 | X |
| | LC T05 | X |
| | LC T06 | X |
| | LC T07 | X |
| | LC T08 | X |
| | LC T09 | X |
| Constant Light | LC T10 | R |
| | LC T11 | TVAAPSVFIFPPSDEQLK |
| | LC T12 | SGTASVVCLLNNFYPR |
| | LC T13 | EAK |
| | LC T14 | VQWK |
| | LC T15 | VDNALQSGNSQESVTEQDSK |
| | LC T16 | DSTYSLSSTLTLSK |
| | LC T17 | ADYEK |
| | LC T18 | HK |
| | LC T19 | VYACEVTHQGLSSPVTK |
| | LC T20 | SFNR |
| | LC T21 | GEC |
| Variable Heavy | HC T01 | X |
| | HC T02 | X |
| | HC T03 | X |
| | HC T04 | X |
| | HC T05 | X |
| | HC T06 | X |
| | HC T07 | X |
| | HC T08 | X |
| | HC T09 | X |
| | HC T10 | X |
| Constant Heavy 1 | HC T11 | GPSVFPLAPCSR |
| | HC T12 | STSESTAALGCLVK |
| | HC T13 | DYFPEPVTVSWNSGALTSGVHTFPAVLQSSGLYSLSSVVTVPSSSLGTK |
| | HC T14 | TYTCNVDHKPSNTK |
| | HC T15 | VDK |
| | HC T16 | R |
| Constant Heavy 1 / Hinge | HC T17 | VESK |
| Hinge/ Constant Heavy 2 | HC T18 | YGPPCPPCPAPEFLGGPSVFLFPPKPK |
| Constant Heavy 2 | HC T19 | DTLMISR |
| | HC T20 | TPEVTCVVVDVSQEDPEVQFNWYVDGVEVHNAK |
| | HC T21 | TKPR |
| | HC T22 | EEQFNSTYR |
| | HC T23 | VVSVLTVLHQDWLNGK |
| | HC T24 | EYK |
| | HC T25 | CK |
| | HC T26 | VSNK |
| | HC T27 | GLPSSIEK |
| | HC T28 | TISK |
| Constant Heavy 2 /Constant Heavy 3 | HC T29 | AK |
| Constant Heavy 3 | HC T30 | GQPR |
| | HC T31 | EPQVYTLPPSQEEMTK |
| | HC T32 | NQVSLTCLVK |
| | HC T33 | GFYPSDIAVEWESNGQPENNYK |
| | HC T34 | TTPPVLDSDGSFFLYSR |
| | HC T35 | LTVDK |
| | HC T36 | SR |
| | HC T37 | WQEGNVFSCSVMHEALHNHYTQK |
| | HC T38 | SLSLSLGK |
Supplemental Table S1 – Theoretical peptides from the in-silico tryptic digest of mAbA.

## Slide 2
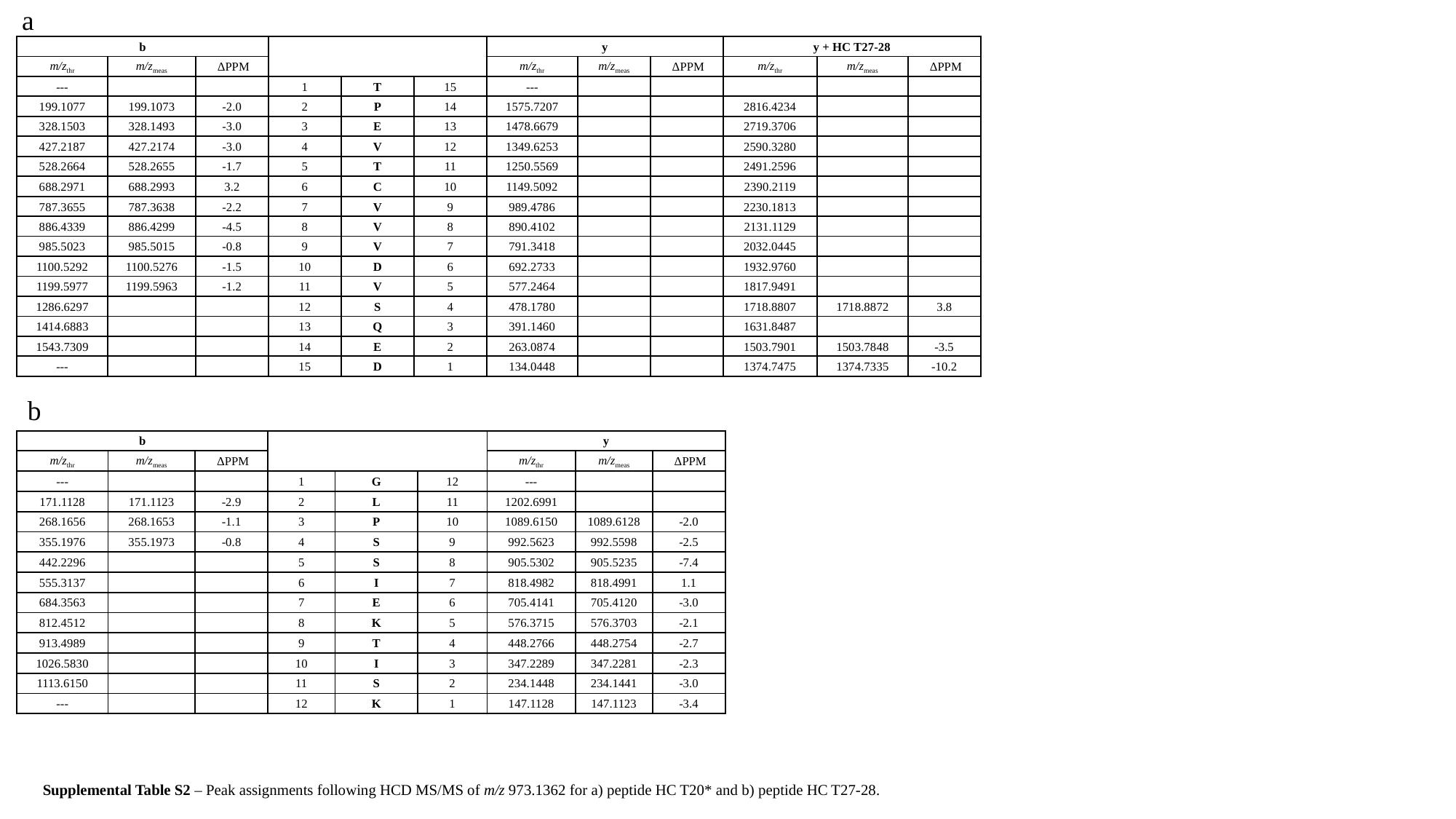

a
| b | | | | | | y | | | y + HC T27-28 | | |
| --- | --- | --- | --- | --- | --- | --- | --- | --- | --- | --- | --- |
| m/zthr | m/zmeas | ΔPPM | | | | m/zthr | m/zmeas | ΔPPM | m/zthr | m/zmeas | ΔPPM |
| --- | | | 1 | T | 15 | --- | | | | | |
| 199.1077 | 199.1073 | -2.0 | 2 | P | 14 | 1575.7207 | | | 2816.4234 | | |
| 328.1503 | 328.1493 | -3.0 | 3 | E | 13 | 1478.6679 | | | 2719.3706 | | |
| 427.2187 | 427.2174 | -3.0 | 4 | V | 12 | 1349.6253 | | | 2590.3280 | | |
| 528.2664 | 528.2655 | -1.7 | 5 | T | 11 | 1250.5569 | | | 2491.2596 | | |
| 688.2971 | 688.2993 | 3.2 | 6 | C | 10 | 1149.5092 | | | 2390.2119 | | |
| 787.3655 | 787.3638 | -2.2 | 7 | V | 9 | 989.4786 | | | 2230.1813 | | |
| 886.4339 | 886.4299 | -4.5 | 8 | V | 8 | 890.4102 | | | 2131.1129 | | |
| 985.5023 | 985.5015 | -0.8 | 9 | V | 7 | 791.3418 | | | 2032.0445 | | |
| 1100.5292 | 1100.5276 | -1.5 | 10 | D | 6 | 692.2733 | | | 1932.9760 | | |
| 1199.5977 | 1199.5963 | -1.2 | 11 | V | 5 | 577.2464 | | | 1817.9491 | | |
| 1286.6297 | | | 12 | S | 4 | 478.1780 | | | 1718.8807 | 1718.8872 | 3.8 |
| 1414.6883 | | | 13 | Q | 3 | 391.1460 | | | 1631.8487 | | |
| 1543.7309 | | | 14 | E | 2 | 263.0874 | | | 1503.7901 | 1503.7848 | -3.5 |
| --- | | | 15 | D | 1 | 134.0448 | | | 1374.7475 | 1374.7335 | -10.2 |
b
| b | | | | | | y | | |
| --- | --- | --- | --- | --- | --- | --- | --- | --- |
| m/zthr | m/zmeas | ΔPPM | | | | m/zthr | m/zmeas | ΔPPM |
| --- | | | 1 | G | 12 | --- | | |
| 171.1128 | 171.1123 | -2.9 | 2 | L | 11 | 1202.6991 | | |
| 268.1656 | 268.1653 | -1.1 | 3 | P | 10 | 1089.6150 | 1089.6128 | -2.0 |
| 355.1976 | 355.1973 | -0.8 | 4 | S | 9 | 992.5623 | 992.5598 | -2.5 |
| 442.2296 | | | 5 | S | 8 | 905.5302 | 905.5235 | -7.4 |
| 555.3137 | | | 6 | I | 7 | 818.4982 | 818.4991 | 1.1 |
| 684.3563 | | | 7 | E | 6 | 705.4141 | 705.4120 | -3.0 |
| 812.4512 | | | 8 | K | 5 | 576.3715 | 576.3703 | -2.1 |
| 913.4989 | | | 9 | T | 4 | 448.2766 | 448.2754 | -2.7 |
| 1026.5830 | | | 10 | I | 3 | 347.2289 | 347.2281 | -2.3 |
| 1113.6150 | | | 11 | S | 2 | 234.1448 | 234.1441 | -3.0 |
| --- | | | 12 | K | 1 | 147.1128 | 147.1123 | -3.4 |
Supplemental Table S2 – Peak assignments following HCD MS/MS of m/z 973.1362 for a) peptide HC T20* and b) peptide HC T27-28.

## Slide 3
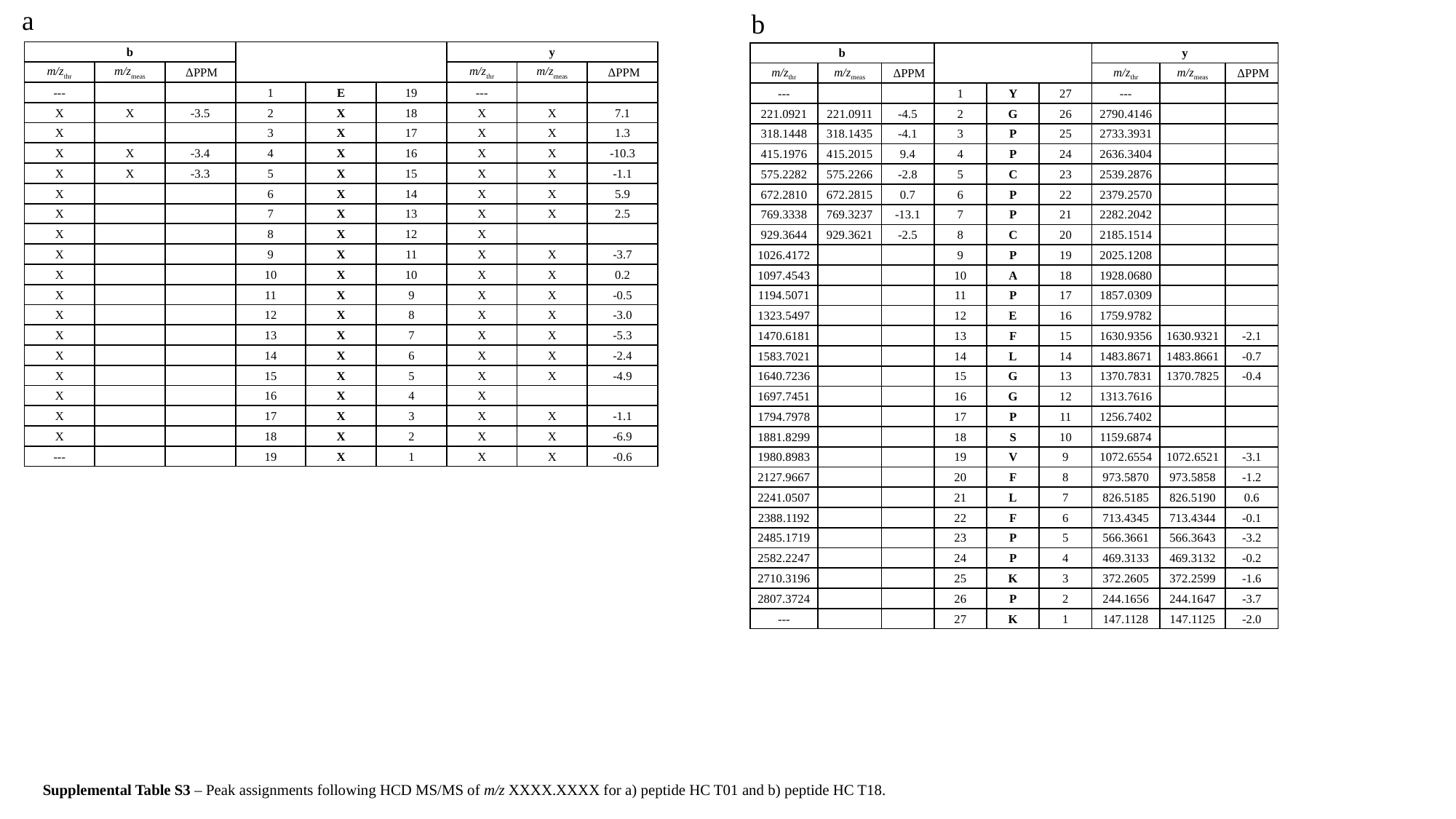

a
b
| b | | | | | | y | | |
| --- | --- | --- | --- | --- | --- | --- | --- | --- |
| m/zthr | m/zmeas | ΔPPM | | | | m/zthr | m/zmeas | ΔPPM |
| --- | | | 1 | E | 19 | --- | | |
| X | X | -3.5 | 2 | X | 18 | X | X | 7.1 |
| X | | | 3 | X | 17 | X | X | 1.3 |
| X | X | -3.4 | 4 | X | 16 | X | X | -10.3 |
| X | X | -3.3 | 5 | X | 15 | X | X | -1.1 |
| X | | | 6 | X | 14 | X | X | 5.9 |
| X | | | 7 | X | 13 | X | X | 2.5 |
| X | | | 8 | X | 12 | X | | |
| X | | | 9 | X | 11 | X | X | -3.7 |
| X | | | 10 | X | 10 | X | X | 0.2 |
| X | | | 11 | X | 9 | X | X | -0.5 |
| X | | | 12 | X | 8 | X | X | -3.0 |
| X | | | 13 | X | 7 | X | X | -5.3 |
| X | | | 14 | X | 6 | X | X | -2.4 |
| X | | | 15 | X | 5 | X | X | -4.9 |
| X | | | 16 | X | 4 | X | | |
| X | | | 17 | X | 3 | X | X | -1.1 |
| X | | | 18 | X | 2 | X | X | -6.9 |
| --- | | | 19 | X | 1 | X | X | -0.6 |
| b | | | | | | y | | |
| --- | --- | --- | --- | --- | --- | --- | --- | --- |
| m/zthr | m/zmeas | ΔPPM | | | | m/zthr | m/zmeas | ΔPPM |
| --- | | | 1 | Y | 27 | --- | | |
| 221.0921 | 221.0911 | -4.5 | 2 | G | 26 | 2790.4146 | | |
| 318.1448 | 318.1435 | -4.1 | 3 | P | 25 | 2733.3931 | | |
| 415.1976 | 415.2015 | 9.4 | 4 | P | 24 | 2636.3404 | | |
| 575.2282 | 575.2266 | -2.8 | 5 | C | 23 | 2539.2876 | | |
| 672.2810 | 672.2815 | 0.7 | 6 | P | 22 | 2379.2570 | | |
| 769.3338 | 769.3237 | -13.1 | 7 | P | 21 | 2282.2042 | | |
| 929.3644 | 929.3621 | -2.5 | 8 | C | 20 | 2185.1514 | | |
| 1026.4172 | | | 9 | P | 19 | 2025.1208 | | |
| 1097.4543 | | | 10 | A | 18 | 1928.0680 | | |
| 1194.5071 | | | 11 | P | 17 | 1857.0309 | | |
| 1323.5497 | | | 12 | E | 16 | 1759.9782 | | |
| 1470.6181 | | | 13 | F | 15 | 1630.9356 | 1630.9321 | -2.1 |
| 1583.7021 | | | 14 | L | 14 | 1483.8671 | 1483.8661 | -0.7 |
| 1640.7236 | | | 15 | G | 13 | 1370.7831 | 1370.7825 | -0.4 |
| 1697.7451 | | | 16 | G | 12 | 1313.7616 | | |
| 1794.7978 | | | 17 | P | 11 | 1256.7402 | | |
| 1881.8299 | | | 18 | S | 10 | 1159.6874 | | |
| 1980.8983 | | | 19 | V | 9 | 1072.6554 | 1072.6521 | -3.1 |
| 2127.9667 | | | 20 | F | 8 | 973.5870 | 973.5858 | -1.2 |
| 2241.0507 | | | 21 | L | 7 | 826.5185 | 826.5190 | 0.6 |
| 2388.1192 | | | 22 | F | 6 | 713.4345 | 713.4344 | -0.1 |
| 2485.1719 | | | 23 | P | 5 | 566.3661 | 566.3643 | -3.2 |
| 2582.2247 | | | 24 | P | 4 | 469.3133 | 469.3132 | -0.2 |
| 2710.3196 | | | 25 | K | 3 | 372.2605 | 372.2599 | -1.6 |
| 2807.3724 | | | 26 | P | 2 | 244.1656 | 244.1647 | -3.7 |
| --- | | | 27 | K | 1 | 147.1128 | 147.1125 | -2.0 |
Supplemental Table S3 – Peak assignments following HCD MS/MS of m/z XXXX.XXXX for a) peptide HC T01 and b) peptide HC T18.

## Slide 4
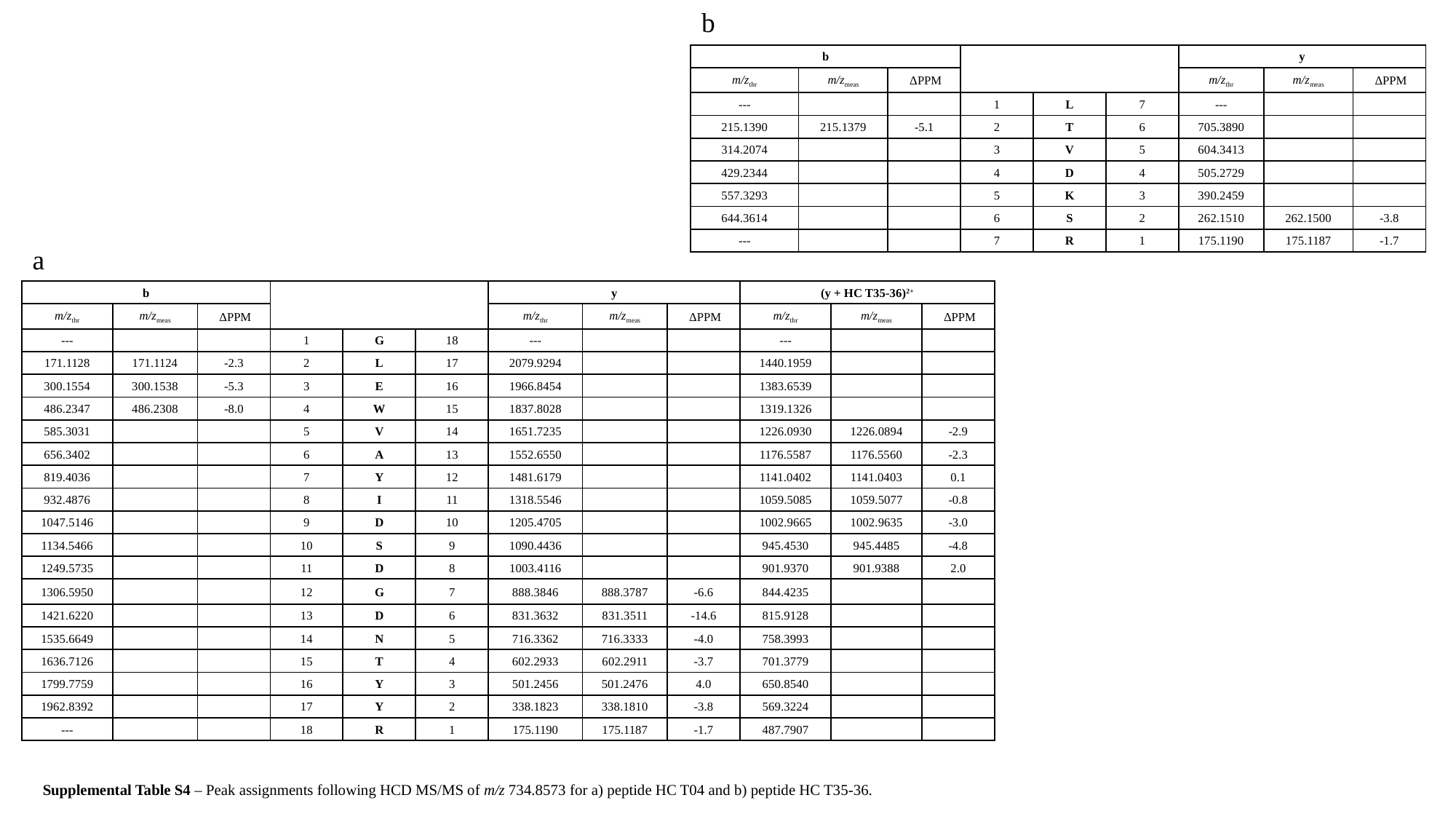

b
| b | | | | | | y | | |
| --- | --- | --- | --- | --- | --- | --- | --- | --- |
| m/zthr | m/zmeas | ΔPPM | | | | m/zthr | m/zmeas | ΔPPM |
| --- | | | 1 | L | 7 | --- | | |
| 215.1390 | 215.1379 | -5.1 | 2 | T | 6 | 705.3890 | | |
| 314.2074 | | | 3 | V | 5 | 604.3413 | | |
| 429.2344 | | | 4 | D | 4 | 505.2729 | | |
| 557.3293 | | | 5 | K | 3 | 390.2459 | | |
| 644.3614 | | | 6 | S | 2 | 262.1510 | 262.1500 | -3.8 |
| --- | | | 7 | R | 1 | 175.1190 | 175.1187 | -1.7 |
a
| b | | | | | | y | | | (y + HC T35-36)2+ | | |
| --- | --- | --- | --- | --- | --- | --- | --- | --- | --- | --- | --- |
| m/zthr | m/zmeas | ΔPPM | | | | m/zthr | m/zmeas | ΔPPM | m/zthr | m/zmeas | ΔPPM |
| --- | | | 1 | G | 18 | --- | | | --- | | |
| 171.1128 | 171.1124 | -2.3 | 2 | L | 17 | 2079.9294 | | | 1440.1959 | | |
| 300.1554 | 300.1538 | -5.3 | 3 | E | 16 | 1966.8454 | | | 1383.6539 | | |
| 486.2347 | 486.2308 | -8.0 | 4 | W | 15 | 1837.8028 | | | 1319.1326 | | |
| 585.3031 | | | 5 | V | 14 | 1651.7235 | | | 1226.0930 | 1226.0894 | -2.9 |
| 656.3402 | | | 6 | A | 13 | 1552.6550 | | | 1176.5587 | 1176.5560 | -2.3 |
| 819.4036 | | | 7 | Y | 12 | 1481.6179 | | | 1141.0402 | 1141.0403 | 0.1 |
| 932.4876 | | | 8 | I | 11 | 1318.5546 | | | 1059.5085 | 1059.5077 | -0.8 |
| 1047.5146 | | | 9 | D | 10 | 1205.4705 | | | 1002.9665 | 1002.9635 | -3.0 |
| 1134.5466 | | | 10 | S | 9 | 1090.4436 | | | 945.4530 | 945.4485 | -4.8 |
| 1249.5735 | | | 11 | D | 8 | 1003.4116 | | | 901.9370 | 901.9388 | 2.0 |
| 1306.5950 | | | 12 | G | 7 | 888.3846 | 888.3787 | -6.6 | 844.4235 | | |
| 1421.6220 | | | 13 | D | 6 | 831.3632 | 831.3511 | -14.6 | 815.9128 | | |
| 1535.6649 | | | 14 | N | 5 | 716.3362 | 716.3333 | -4.0 | 758.3993 | | |
| 1636.7126 | | | 15 | T | 4 | 602.2933 | 602.2911 | -3.7 | 701.3779 | | |
| 1799.7759 | | | 16 | Y | 3 | 501.2456 | 501.2476 | 4.0 | 650.8540 | | |
| 1962.8392 | | | 17 | Y | 2 | 338.1823 | 338.1810 | -3.8 | 569.3224 | | |
| --- | | | 18 | R | 1 | 175.1190 | 175.1187 | -1.7 | 487.7907 | | |
Supplemental Table S4 – Peak assignments following HCD MS/MS of m/z 734.8573 for a) peptide HC T04 and b) peptide HC T35-36.

## Slide 5
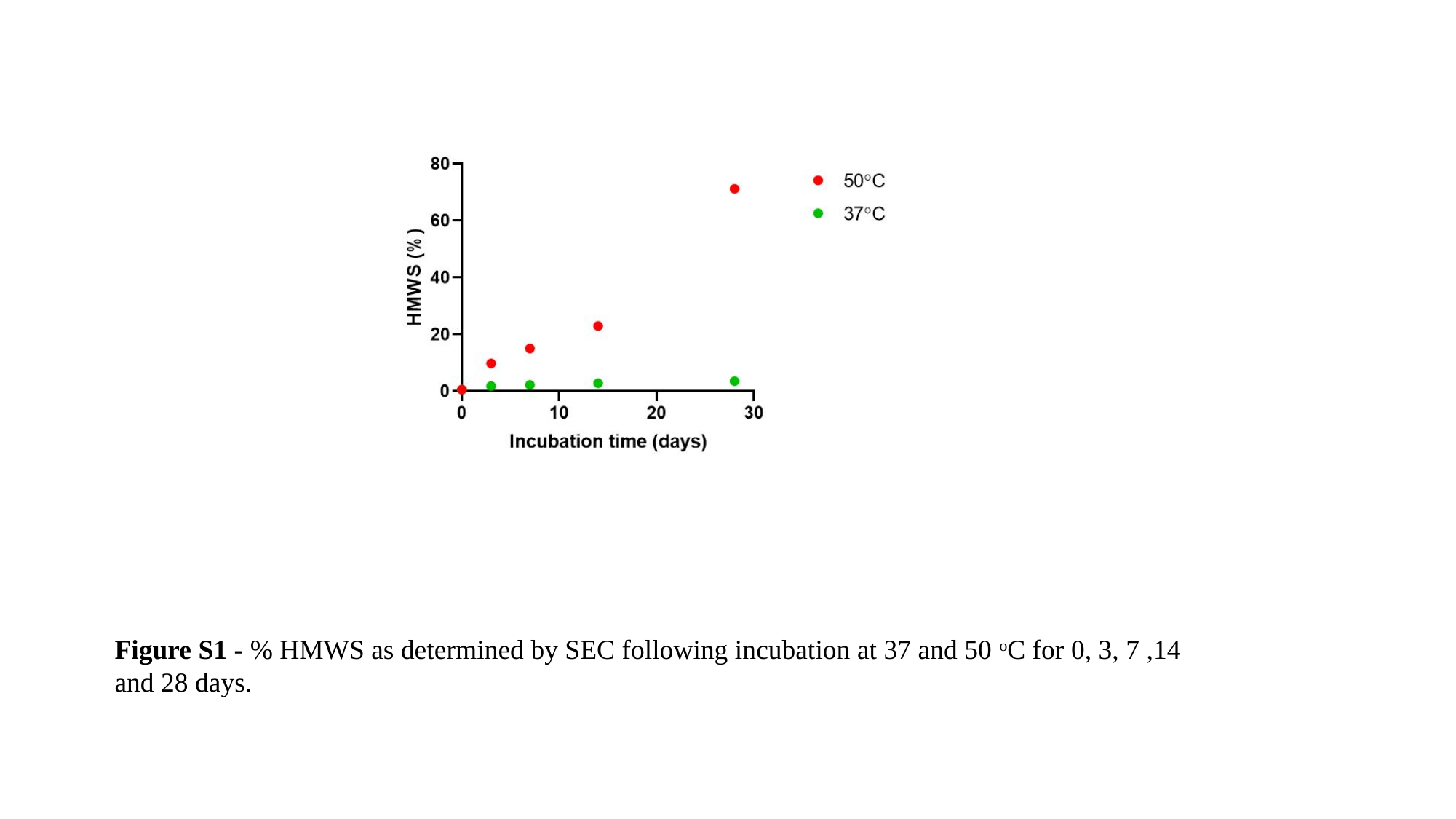

Figure S1 - % HMWS as determined by SEC following incubation at 37 and 50 oC for 0, 3, 7 ,14 and 28 days.

## Slide 6
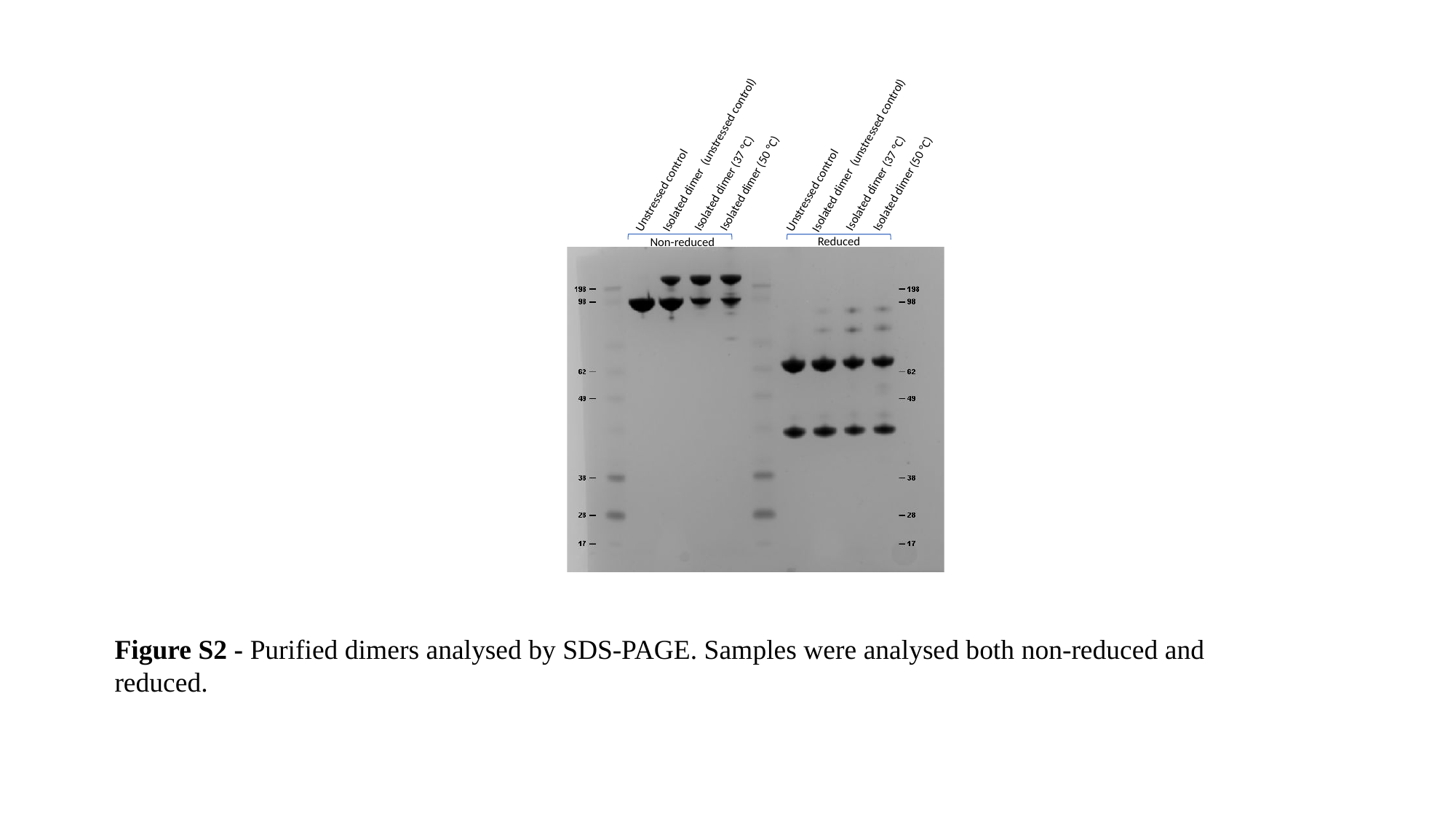

Isolated dimer (unstressed control)
Isolated dimer (unstressed control)
Isolated dimer (50 °C)
Isolated dimer (37 °C)
Isolated dimer (37 °C)
Isolated dimer (50 °C)
Unstressed control
Unstressed control
Reduced
Non-reduced
Figure S2 - Purified dimers analysed by SDS-PAGE. Samples were analysed both non-reduced and reduced.

## Slide 7
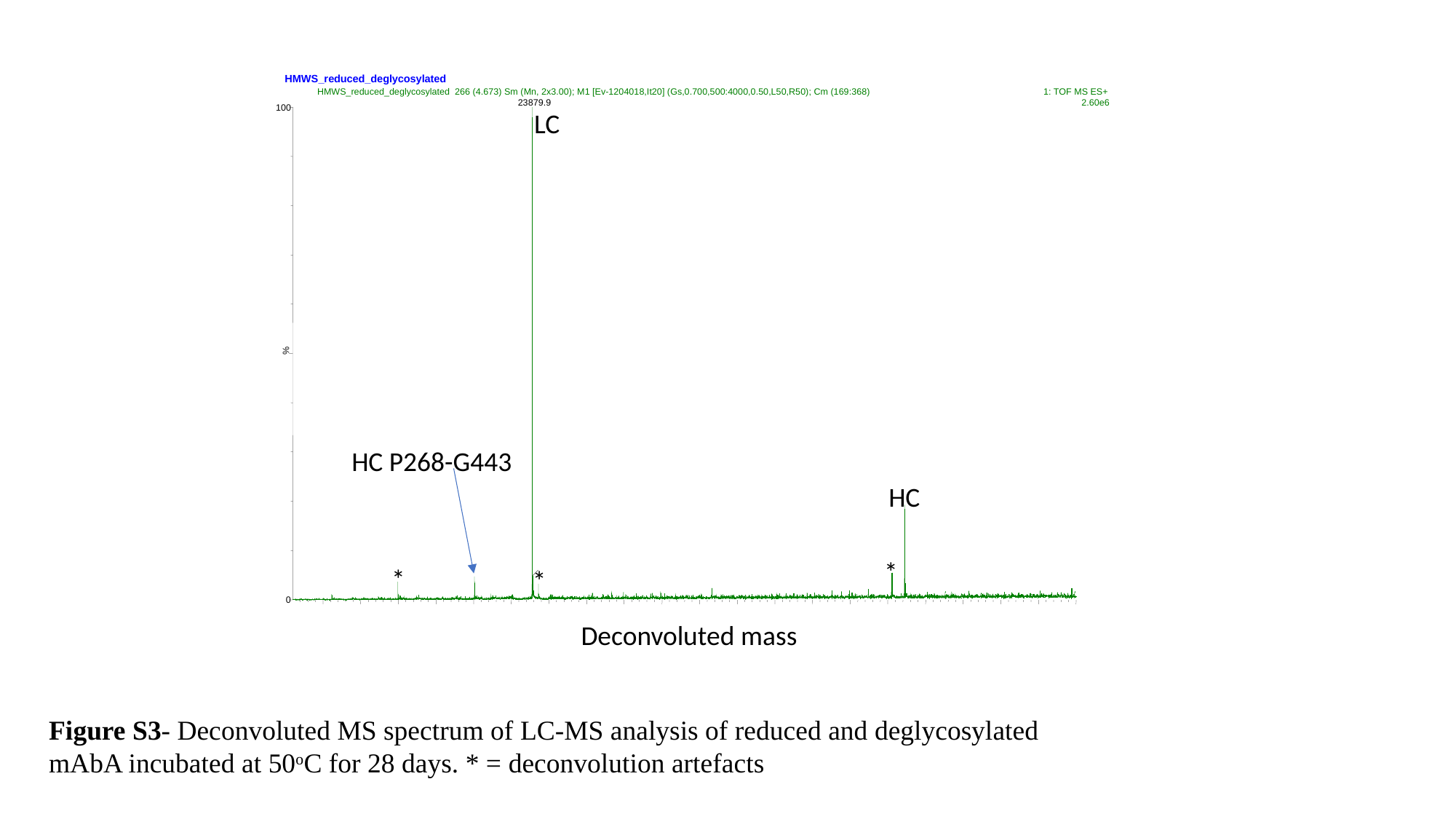

HMWS_reduced_deglycosylated
HMWS_reduced_deglycosylated 266 (4.673) Sm (Mn, 2x3.00); M1 [Ev-1204018,It20] (Gs,0.700,500:4000,0.50,L50,R50); Cm (169:368)
1: TOF MS ES+
23879.9
2.60e6
LC
100
%
HC P268-G443
HC
*
*
*
0
Deconvoluted mass
Figure S3- Deconvoluted MS spectrum of LC-MS analysis of reduced and deglycosylated mAbA incubated at 50oC for 28 days. * = deconvolution artefacts

## Slide 8
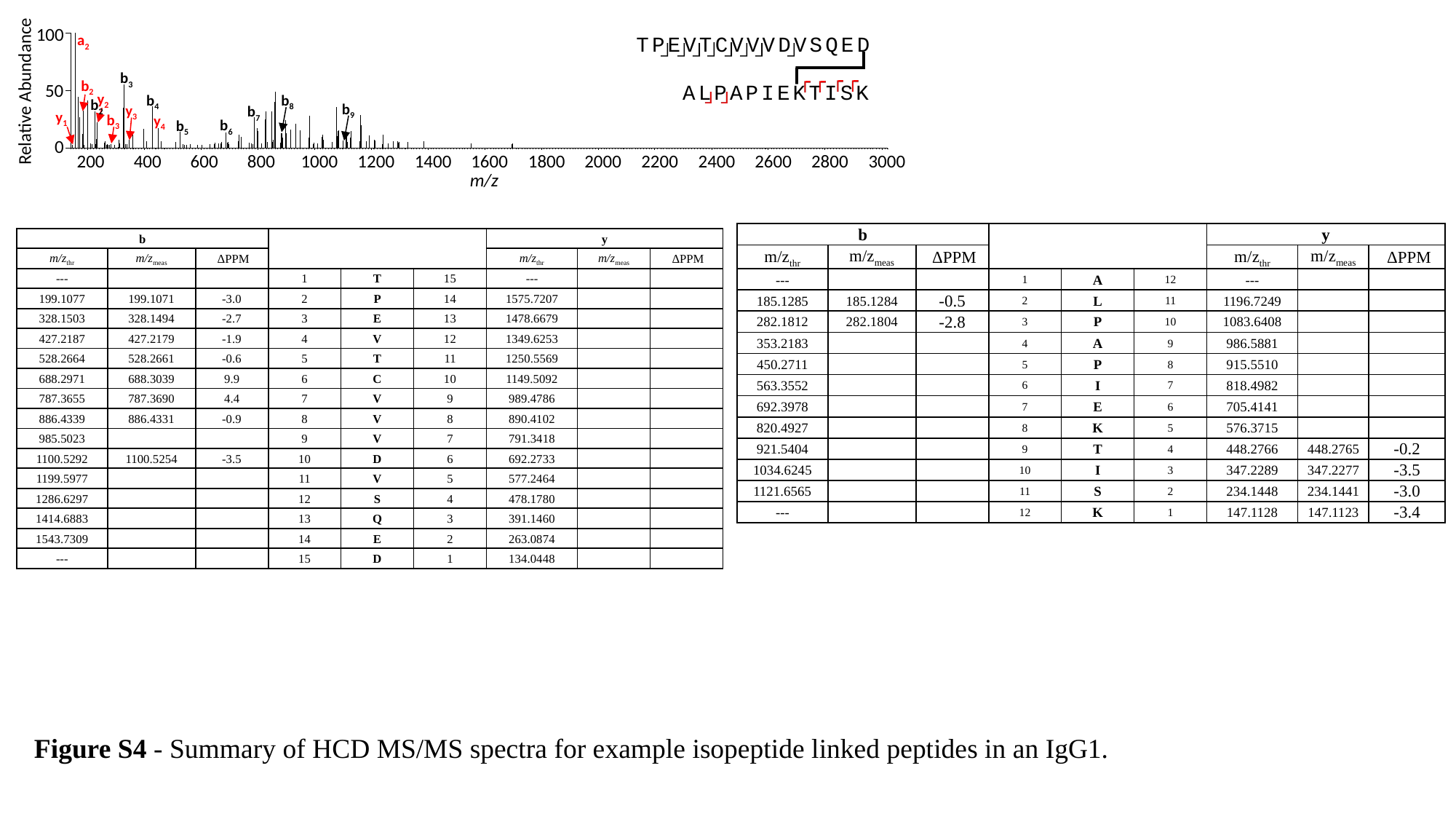

100
a2
TPEVTCVVVDVSQED
b3
b2
ALPAPIEKTISK
50
Relative Abundance
y2
b4
b8
b2
b9
y3
b7
y1
b3
y4
b6
b5
0
200
400
600
800
1000
1200
1400
1600
1800
2000
2200
2400
2600
2800
3000
m/z
| b | | | | | | y | | |
| --- | --- | --- | --- | --- | --- | --- | --- | --- |
| m/zthr | m/zmeas | ΔPPM | | | | m/zthr | m/zmeas | ΔPPM |
| --- | | | 1 | A | 12 | --- | | |
| 185.1285 | 185.1284 | -0.5 | 2 | L | 11 | 1196.7249 | | |
| 282.1812 | 282.1804 | -2.8 | 3 | P | 10 | 1083.6408 | | |
| 353.2183 | | | 4 | A | 9 | 986.5881 | | |
| 450.2711 | | | 5 | P | 8 | 915.5510 | | |
| 563.3552 | | | 6 | I | 7 | 818.4982 | | |
| 692.3978 | | | 7 | E | 6 | 705.4141 | | |
| 820.4927 | | | 8 | K | 5 | 576.3715 | | |
| 921.5404 | | | 9 | T | 4 | 448.2766 | 448.2765 | -0.2 |
| 1034.6245 | | | 10 | I | 3 | 347.2289 | 347.2277 | -3.5 |
| 1121.6565 | | | 11 | S | 2 | 234.1448 | 234.1441 | -3.0 |
| --- | | | 12 | K | 1 | 147.1128 | 147.1123 | -3.4 |
| b | | | | | | y | | |
| --- | --- | --- | --- | --- | --- | --- | --- | --- |
| m/zthr | m/zmeas | ΔPPM | | | | m/zthr | m/zmeas | ΔPPM |
| --- | | | 1 | T | 15 | --- | | |
| 199.1077 | 199.1071 | -3.0 | 2 | P | 14 | 1575.7207 | | |
| 328.1503 | 328.1494 | -2.7 | 3 | E | 13 | 1478.6679 | | |
| 427.2187 | 427.2179 | -1.9 | 4 | V | 12 | 1349.6253 | | |
| 528.2664 | 528.2661 | -0.6 | 5 | T | 11 | 1250.5569 | | |
| 688.2971 | 688.3039 | 9.9 | 6 | C | 10 | 1149.5092 | | |
| 787.3655 | 787.3690 | 4.4 | 7 | V | 9 | 989.4786 | | |
| 886.4339 | 886.4331 | -0.9 | 8 | V | 8 | 890.4102 | | |
| 985.5023 | | | 9 | V | 7 | 791.3418 | | |
| 1100.5292 | 1100.5254 | -3.5 | 10 | D | 6 | 692.2733 | | |
| 1199.5977 | | | 11 | V | 5 | 577.2464 | | |
| 1286.6297 | | | 12 | S | 4 | 478.1780 | | |
| 1414.6883 | | | 13 | Q | 3 | 391.1460 | | |
| 1543.7309 | | | 14 | E | 2 | 263.0874 | | |
| --- | | | 15 | D | 1 | 134.0448 | | |
Figure S4 - Summary of HCD MS/MS spectra for example isopeptide linked peptides in an IgG1.
